# Direct protamine activation of human mast cells is MRGPRX2-dependent and is modulated by heparin

**DOI:** 10.1016/j.jpet.2026.104313

**Published:** 2026-03-04

**Authors:** Nithya A. Fernandopulle, Jie Ding, Gavan Francis, Mark D. Hulett, Paul F. Soeding, Lauren T. May, Graham A. Mackay

**Affiliations:** 1 Department of Biochemistry & Pharmacology, The University of Melbourne, Parkville, Victoria, Australia; 2 Department of Biochemistry and Chemistry, La Trobe Institute for Molecular Science, La Trobe University, Bundoora, Victoria, Australia; 3 Department of Anaesthetics and Pain Medicine, The Royal Melbourne Hospital, Parkville, Victoria, Australia; 4 Drug Discovery Biology, Monash Institute of Pharmaceutical Sciences, Monash University, Parkville, Victoria, Australia

**Keywords:** Mast cells, MRGPRX2, Drug hypersensitivity

## Abstract

Protamine is a polybasic drug commonly used in cardiac surgery to chemically inhibit, and thereby reverse, the anticoagulant effect of heparin. However, protamine can trigger severe anaphylaxis, with both IgE-dependent and IgE-independent mechanisms implicated. Recent research has linked the receptor MRGPRX2 (Mas-related G protein-coupled receptor X2) to IgE-independent mast cell activation by polybasic drugs, but its direct interaction with protamine has not been studied. This study investigated the role of MRGPRX2 in protamine-induced mast cell activation and explored how heparin influenced MRGPRX2 activation by protamine and other MRGPRX2 agonists. We used an in vitro cultured human mast cell line, Laboratory of Allergic Diseases 2, that constitutively expresses MRGPRX2. Receptor dependence of agonist-mediated signal transduction was demonstrated using a CRISPR MRGPRX2 knock down Laboratory of Allergic Diseases 2 cell line and a MRGPRX2 inverse-agonist. Mast cell activation was quantified using intracellular calcium mobilization, cellular degranulation, and cytokine release. Protamine-induced a concentration-dependent mast cell activation and was characterized as an unbiased partial agonist at MRGPRX2. Protamine-induced responses were significantly reduced by MRGPRX2 knock down and receptor antagonism. Heparin, which is known to complex with protamine, reduced the mast cell activation induced by protamine, compound 48/80, and LL-37, but not by the other MRGPRX2 agonists assessed. Isothermal titration calorimetry demonstrated that this selectivity relates to ligand ability to bind heparin. Recapitulation of clinically used ratios of protamine:heparin revealed that under these conditions, protamine still triggered effective mast cell activation, highlighting the need for further research to better understand the clinical relevance of MRGPRX2 activation by protamine.

**Significance Statement:**

This study identified protamine, a polybasic drug commonly used in cardiac surgery, as a partial agonist at the MRGPRX2 receptor, and that heparin significantly inhibited the receptor activation by protamine and other endogenous MRGPRX2 agonists. However, the commonly used clinical ratio of protamine:heparin still permitted mast cell activation in vitro.

## 1 Introduction

Protamine is used clinically to reverse the anticoagulant activity of heparin, particularly in cardiac surgery involving cardiopulmonary bypass and in settings of heparin overdose.^1^^,^^2^ Protamine is a positively charged peptide (poly-arginine) that interacts with the negative charge on heparin to form a stable complex. This polyanionic-cationic binding neutralizes the anticoagulant activity of heparin, enabling rapid and safe separation from cardiopulmonary bypass.^1^

Alongside neuromuscular blocking agents and antibiotics, protamine remains a significant cause of anaphylaxis in cardiac surgery, triggering profound cardiac collapse.^3^^,^^4^ Investigations into causative mechanisms of anaphylaxis to protamine have provided evidence for both IgE-dependent and IgE-independent reactions.^5^ IgE-dependent anaphylaxis to protamine has been relatively well studied and risk factors are characterized. High-risk individuals include diabetic patients previously medicated with protamine-zinc-insulin, and infertile or vasectomized men who have developed antisperm antibodies.^4^, ^5^, ^6^ However, the mechanism of non–IgE-mediated anaphylaxis to protamine is not well characterized, and speculation remains as to whether direct mast cell activation is a primary mechanism.^3^^,^^7^

The mast cell receptor MRGPRX2 (Mas-related G protein-coupled receptor X2), has been identified as a possible key player in IgE-independent anaphylaxis.^8^^,^^9^ Agonists at this receptor are known to have polybasic structures and include clinically used drugs such as certain neuromuscular blocking agents, antibiotics, opioids, endogenous peptides such as substance P, and laboratory tools including compound 48/80 and ZINC-3573(R).^10^^,^^11^ The structure of MRGPRX2, a G protein-coupled receptor, in complex with various agonists has recently been determined.^12^^,^^13^ Protamine has been shown to directly activate mast cells,^14^^,^^15^ and given its polybasic structure, we hypothesize these effects would be mediated via MRGPRX2 activation.

This in vitro study evaluated the role of MRGPRX2 in IgE-independent mast cell activation by protamine and determined any modulation of this effect by heparin.

## 2 Materials and methods

### 2.1 Materials

The following products were purchased from Merck (ZINC-3573(R), ciprofloxacin, Triton-X100, Percoll, Hank's buffered salts, sodium bicarbonate, HEPES, glucose, bovine serum albumin (BSA), MgSO4, CaCl2, round and flat bottom 96-well plates, Corning white flat bottom 96-well plates (CLS3922), probenecid, FBS, and glycine. Compound 48/80 was obtained from Enzo Life Sciences or Merck. LL-37 and cortistatin-14 were purchased from Cayman Chemical Company. Clinical grade protamine and heparin were obtained from the Royal Melbourne Hospital pharmacy. NIP-BSA (4-hydroxy-3-iodo-5-nitrophenylacetyl bovine serum albumin) was purchased from Biosearch Technologies. Substance P was synthesized and purified (>95% purity) by Ms. Lin Hsin and Dr. Susan Northfield (Department of Biochemistry and Pharmacology, University of Melbourne, Australia). Eight-well chamber slides were purchased from IBIDI. The following products were purchased from ThermoFisher Scientific; StemPro-34, GlutaMAX, and penicillin (5000 unit/mL)/streptomycin (5000 *μ*g/mL). Human stem cell factor was from Peprotech. FURA-2AM was purchased from Abcam. pNAG (p-nitrophenyl-N-acetyl-β-D-glucosaminide) was purchased from Glycosynth. TMB substrate (3,3′,5,5′-Tetramethylbenzidine) and CCL2 (MCP-1) cytokine ELISA kit were purchased from BD Biosciences. Human anti-FcεRIα-APC (clone Cra-1) and anti-MRGPRX2-PE (clone K125H4), along with appropriately labeled isotype controls, were purchased from BioLegend.

### 2.2 Cell culture

Laboratory of Allergic Diseases 2 (LAD2) cells^16^ were kindly provided by Dr. Arnold Kirshenbaum (National Institute of Health). The cells were cultured in StemPro-34 medium supplemented with stem cell factor (100 ng/mL), GlutaMAX (1X) and penicillin (50 units/mL)/streptomycin (50 *μ*g/mL), and maintained in a humidified incubator (5% CO_2_, 37 ˚C).

Human embryonic kidney-293 (HEK-293) cells were maintained in Iscove’s Modified Dulbecco’s Media (IMDM) supplemented with 10% FBS, GlutaMAX (2mM), penicillin (50 units/mL) and streptomycin (50 *μ*g/mL). All cells were tested regularly for the presence of mycoplasma contamination (MycoAlert PLUS Mycoplasma Detection Kit) and found to be negative.

### 2.3 Generation of MRGPRX2 knock down LAD2 cells

MRGPRX2 knock down (KD) LAD2 cells were generated through CRISPR-Cas9 technology and sorted initially on a CD63^low^ population following activation with compound 48/80 (BD fluorescence-activated cell sorting ARIA III; BD Biosciences).^17^ MRGPRX2 low cells were subsequently enriched using an anti-MRGPRX2 antibody.^18^ As in our hands, LAD2 cells are not able to be expanded from clones in simple culture, a mixed polyclonal population was used throughout (MRGPRX2 KD cells).

### 2.4 Quantifying real-time calcium mobilization in LAD2 cells

LAD2 cells were sensitized with human NIP-specific IgE (1–2 *μ*g/mL; conditioned media from Jw8/5/13; ECACC hybridoma collection) for 24–48 hours. The cells were then incubated with FURA2-AM (2 *μ*M) in Hank’s buffered saline solution (HBSS) (1X Hank's buffered salts, sodium bicarbonate (0.14%), HEPES (10 mM), glucose (5.5 mM), BSA (0.05%), MgSO_4_ (0.7 mM), CaCl_2_ (1.8 mM)) supplemented with probenecid (2.5 mM) at a density of 5 × 10^6^ cells/mL for 90 minutes at 37 ˚C with gentle shaking. Cells were then washed and seeded into 96-well plates and, following a 30-second baseline reading, protamine (3.75–30 *μ*g/mL), independently or in premixed complexes with heparin (375–3000 mUSP units/mL), was added to wells and ratiometric recordings of fluorescence emission measurements (excitation 340/380 nm; emission 510 nm) taken every 5 seconds for 6 minutes (FlexStation III; Molecular Devices). Where appropriate, PRISM (GraphPad PRISM version 10) was used to determine peak fluorescence emission values.

### 2.5 Quantification of LAD2 degranulation by β-hexosaminidase release

*β*-hexosaminidase, an enzyme found within intracellular granules of mast cells, is a commonly used quantifiable marker of degranulation. IgE-sensitized LAD2 cells were washed and resuspended in HBSS and seeded into 96-well plates. Cells were incubated with protamine (0.001–15 *μ*g/mL), compound 48/80 (0.001–10 *μ*g/mL), ZINC-3573(R) (5 μM), atracurium (200 *μ*M), ciprofloxacin (50 *μ*g/mL), substance P (1-10 *μ*g/mL) and NIP-BSA (10–100 ng/mL) (independently or in premixed complexes with heparin (0.070–10 USP units/mL)) for 45 minutes. Plates were centrifuged, cell-free supernatant (25 *μ*L) was transferred to a fresh plate, and pNAG substrate (75 *μ*L; 2 mM, in phosphate-citrate buffer pH = 4.5) was added. The plate was subsequently incubated for 120 minutes at 37 °C in an oscillating incubator (70 rpm). The reaction was halted and developed by the addition of glycine (100 *μ*L; 0.4 M; pH = 10.7), and an absorbance reading taken at 405 nm (Multiskan Ascent; ThermoFisher Scientific). The absorbance readings were used to calculate *β*-hexosaminidase release as a percentage of the total *β*-hexosaminidase content (quantified using Triton-X (0.1%) induced cell lysis). The percentage of spontaneous release of *β*-hexosaminidase (that occurs in the absence of an added stimulus) was then subtracted from these values.

### 2.6 Quantification of LAD2 cytokine CCL2 release

IgE-sensitized LAD2 cells were washed and resuspended in serum-free IMDM supplemented with 0.1% BSA, GlutaMAX (2 mM) and penicillin (50 units/mL)/streptomycin (50 *μ*g/mL) and seeded into round bottom 96-well plates (2.5 × 10^5^ cells/mL in 200 *μ*L buffer). The cells were treated overnight (18 hours) with stimuli in a humidified incubator (37 °C and 5% CO_2_). Contents of the wells were mixed and transferred to 1.5-mL microcentrifuge tubes and centrifuged (233 g, 5 minutes) to sediment cells. Cell-free supernatant was then removed and CCL2 quantified by ELISA (methodology as per manufacturer's instructions [BD OptEIA; BD Biosciences]). Absorbance readings were taken at 450 nm (Multiskan Ascent; ThermoFisher Scientific), and absolute concentrations of CCL2 were interpolated from a standard curve run consecutively on the same plate.

### 2.7 Isolation of rat peritoneal mast cells and live cell imaging

Peritoneal cells were obtained from euthanized male Sprague-Dawley rats (9–12 weeks old) by lavage. Animals were obtained from an approved project (Project ID: UoM10236; The University of Melbourne, animal ethics committee) as a secondary use procedure. Rat peritoneal mast cells (RPMCs) were purified using a Percoll density gradient method as previously described.^19^ The isolated cells (>95% purity) were then carefully resuspended in HBSS. A portion of these cells were loaded into an 8-well chamber slide for live cell imaging (SP5 microscope; Leica microsystems). Images were taken every 2 seconds for up to 60 seconds following stimulus addition and compiled into a video using Image J (FIJI; National Institutes of Health). Remaining cells (35,000/well) were seeded into round bottom 96-well plates and activated with the various stimuli and degranulation quantified using the *β*-hexosaminidase assay as above.

### 2.8 Calcium mobilization in MRGPRX2-Gα15-transfected HEK293 cells

HEK-293 cells were seeded in 6-well plates at 800,000 cells/well in transfection medium (IMDM supplemented with 1% FBS and GlutaMAX) and cultured overnight. Cells were transfected with a human MRGPRX2 (Genscript) and human G*α*15 (cDNA Resource Center) constructs (1.25 *μ*g/well), using lipofectamine 3000. The latter construct was used to enhance MRGPRX2-mediated signaling to calcium mobilization. MOCK transfected cells were treated with the transfection reagents, without plasmid DNA. Five hours posttransfection, the transfection medium was replaced with fresh medium. Forty-eight hours later, cells were harvested by trypsinization for calcium mobilization and flow cytometry. Flow cytometry analyses were conducted to confirm the surface MRGPRX2 expression using an anti-human-MRGPRX2 antibody (data not shown). Suspension MRGPRX2-G*α*15-transfected HEK293 cells were washed and incubated with Fura-2 AM (4 *μ*M) for 60 minutes at 37 °C in an oscillating incubator protected from light. Fura-2 AM-loaded cells were then washed and plated into flat bottom 96-well plates at a density of 100,000 cells/well. Stimuli were added after 30 seconds of baseline readings and the assay was run for 4 minutes, with readings taken every 5 seconds again using a FlexStation III as described above.

### 2.9 BRET-based G protein activation assay

HEK293 cells were seeded in a 6-well plate (600,000 cells/well) in transfection medium and incubated in a humidified incubator at 37 °C overnight. Cells were then cotransfected with a human MRGPRX2 construct (100 ng/well) and BRET-based human Gq-CASE protein activity sensors^20^ (500 ng/well; Addgene) using lipofectamine 3000 according to the manufacturer's instructions. Five hours posttransfection, the medium was replaced. After 48 hours, cells were harvested for G protein activation assays.

Briefly, cells were washed, resuspended in HBSS buffer (145 mM NaCl, 5 mM KCl, 1.3 mM CaCl_2_, 1 mM MgSO_4_, 10 mM HEPES, 2 mM sodium pyruvate, 1.5 mM NaHCO_3_, 10 mM D-glucose; pH = 7.45) supplemented with 0.1% BSA and plated at a density of 40,000 cells/well in Corning 96-well white cell culture plates. Cells were then incubated at 37 °C for 1 hour prior to addition of Nano-Glo Luciferase assay substrate, Furimazine (Promega). Sequential filtered light emissions were recorded using a CLARIOstar plate reader (BMG Labtech) using 450-nm (40 nm bandpass) and 535-nm (30 nm bandpass) filters for 50 cycles (58 seconds/cycle), and cells were stimulated following 10 cycles of baseline readings. BRET ratios were calculated by the 535-nm emission (acceptor) divided by the 450-nm emission (donor). Baselines were normalized by subtracting the BRET ratio of vehicle-treated cell samples and the BRET ratio of the first 10 cycles of baseline readings.

### 2.10 Characterization of protamine/MRGPRX2 pharmacology

Global analysis using GraphPad PRISM version 10 was conducted on the group data for the MRGPRX2 agonist compound 48/80 and protamine for stimulating calcium mobilization, degranulation, and cytokine (CCL2) release. Molar concentrations required for the calculations in this analysis were based on average molecular weights of the polymeric drugs, compound 48/80 (500 Da^21^) and protamine (4,500 Da^22^). Data were normalized to baseline and compared to the maximal response of the well-characterized full agonist, compound 48/80. Data were fitted to a 4-parameter nonlinear regression to estimate pEC_50_ and E_max_ values. The Black–Leff operational model of partial agonism was fitted using compound 48/80 as the full agonist to derive pK_A_ and Log(t) values for protamine.^23^ Biased agonism of MRGPRX2 was evaluated, as described previously,^24^ using a reparameterization of the Black–Leff operational model. To quantify signaling bias, Log (t/K_A_) values (logarithmic transduction ratios) were calculated as described previously.^25^ Transduction ratios were then normalized to the reference agonist, compound 48/80, and a reference pathway, in this case, calcium mobilization to derive Log(bias) values (ΔΔ Log (t/K_A_)).

### 2.11 Isothermal titration calorimetry

Syringe titrations were performed using the MicroCal iTC200 System (GE Healthcare) at 25 °C (750 rpm) in water (pH = 7.2). Into the sample cell containing heparin (280 *μ*L; 2, 4, 16 USP units/mL), protamine (200 *μ*g/mL), LL37 (200 *μ*M), and substance P (80 *μ*M) were titrated. An initial ligand injection of 0.4 *μ*L was made after 60 seconds, followed by 19 injections of 2 *μ*L per 180 seconds. The initial injection was excluded from data analysis. Appropriate buffer titrant controls were conducted. Data analyses were performed using MicroCal Origin version 7.0 software and fitted using a 1-site binding model. Experiments were repeated 3 times with representative primary data shown. An average molecular weight of heparin (13,500 Da), protamine (9,936.5 Da) and for heparin a standard conversion (180 USP units/mg) were used to estimate binding thermodynamics and affinity.

### 2.12 Data presentation and statistical analysis

Except where otherwise described, data sets are expressed as means ± SEM of N experiments and graphs drawn using GraphPad PRISM (version 10). Concentrations are presented in g/mL except in the Black–Leff biased agonism analysis calculations where molar concentrations were estimated based on average molecular weights. Statistical significance was determined using one-way ANOVA with Dunnett’s multiple comparison post hoc test or two-way ANOVA with Bonferroni multiple comparison post hoc test (version 10, GraphPad PRISM). *P* values are as indicated in figure legends.

## 3 Results

### 3.1 Role of MRGPRX2 in protamine-mediated mast cell activation

**Fig. 1:**
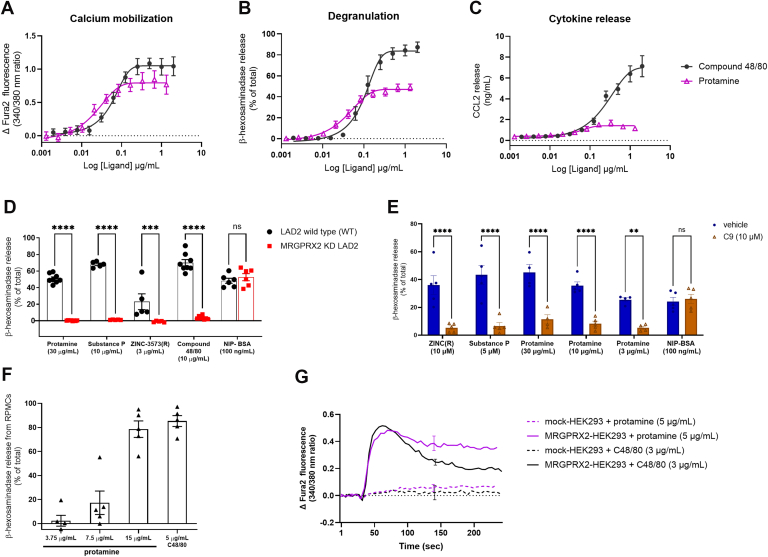
Protamine induces LAD2 mast cell activation via MRGPRX2. LAD2 wild type (LAD2 WT) cells were treated with a concentration range of protamine and the known MRGPRX2 agonist, compound 48/80, to quantify mast cell activation via (A) calcium mobilization, (B) degranulation, and (C) cytokine CCL2 release. (D**)** Degranulation was quantified using *β*-hexosaminidase release in LAD2 WT and LAD2 MRGPRX2 KD cells exposed to protamine (30 *μ*g/mL), compound 48/80 (10 μg/mL), substance P (13 *μ*g/mL), ZINC-3573(R) (3 *μ*g/mL), and NIP-BSA (100 ng/mL). N ≥ 5, mean ± SEM. (E) Degranulation of LAD2 cells following exposure to ZINC-3573(R) (10 μM), substance P (5 μM), protamine (30, 10, 3 μg/mL) and NIP-BSA (100 ng/mL) in the absence and presence of the MRGPRX2 antagonist C9 (10 *μ*M). (F) RPMCs were isolated from male Sprague-Dawley rats using Percoll gradient separation, and stimulated with protamine (15, 7.5, 3.75 *μ*g/mL) and compound 48/80 (5 *μ*g/mL). Degranulation was quantified using *β*-hexosaminidase release. N = 5, mean ± SEM. (G) HEK-293 cells were transiently transfected with MRGPRX2 or empty vector. MRGPRX2-mediated activation was quantified by calcium mobilization. N = 3–4, mean ± SEM. Relevant statistical analysis was conducted using a two-way ANOVA model with Bonferroni multiple comparison post hoc test. ∗∗*P* < .01, ∗∗∗*P* < .001, ∗∗∗∗*P* < .0001.

Protamine-induced a concentration-dependent activation of LAD2 WT mast cell signaling (as quantified by calcium mobilization, degranulation, and cytokine (CCL2) release) (Fig. 1, A–C). In all measured responses, compared to compound 48/80, protamine consistently demonstrated a lowered maximum efficacy, and therefore acted as a partial agonist (Supplemental Table 1). Mast cell degranulation in the presence of protamine was strongly attenuated in the MRGPRX2 knock down LAD2 cells. Similarly, MRGPRX2 KD LAD2 cells abolished the response of well-described MRGPRX2 agonists compound 48/80, substance P, and ZINC-3573(R) (Fig. 1D). Furthermore, antagonism of MRGPRX2 with C9 ^12^, significantly reduced LAD2 WT degranulation induced by protamine, compound 48/80, ZINC-3573(R), and substance P (Fig. 1E). In contrast, the IgE-dependent mast cell activation following exposure LAD2 cells to NIP-BSA was unchanged by MRGPRX2 KD and C9 antagonism (Fig. 1, D and E).

Quantification of protamine affinity, Log(K_A_) and efficacy, Log(t), using the Black–Leff operational model, confirmed protamine was a partial agonist (Supplemental Table 1). These analyses highlighted that MRGPRX2 had the highest coupling efficiency for calcium mobilization, followed by degranulation and then cytokine release, reflected by the change in compound 48/80 potency and protamine maximal effect, Emax and operational efficacy, Log(t) (Fig. 1, A–C; Supplemental Table 1). Given the large changes in protamine maximal response across signaling pathways, additional analysis of biased agonism was performed using the modified Black–Leff operational model.^25^ These analyses revealed that protamine produced proportionate activation across pathways, relative to the full agonist, compound 48/80, with agonists showing no significant difference in coupling efficacy across the different pathways measured (Supplemental Table 1). As such, using the well characterized agonist compound 48/80 as the comparator, protamine can be classified as a partial but unbiased MRGPRX2 agonist.

MRGPRX2-mediated cell activation by protamine was also examined using purified RPMCs (that express the rat homologue of MRGPRX2, Mrgb3) and HEK-293 cells transiently transfected with MRGPRX2. Like compound 48/80 (5 *μ*g/mL), protamine (15 *μ*g/mL) induced significant degranulation of RPMCs (Fig. 1F, Supplemental Video 1A). In HEK-293 cells (Fig. 1G), protamine-induced calcium mobilization in MRGPRX2 transfected cells, but not in mock transfected cells. In keeping with the calcium mobilization data, a BRET-based G protein activation assay showed that MRGPRX2 exposure to protamine, similar to compound 48/80, activated Gq signaling (Supplemental Fig. 1).

### 3.2 Effect of heparin on protamine-induced MRGPRX2 activation

**Fig. 2:**
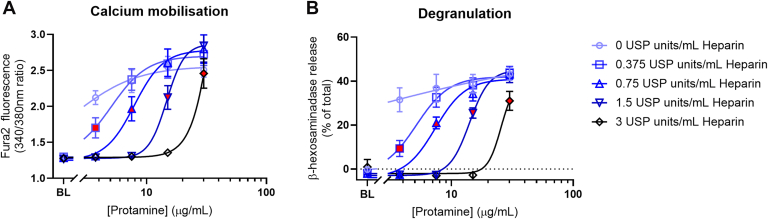
Neutralization of protamine-induced mast cell activation by heparin. LAD2 mast cells were exposed to premixed complexes of protamine (3.75, 7.5, 15, 30 *μ*g/mL) and heparin (0.375, 0.75, 1.5, 3 USP units/mL). Measurements in the absence of protamine are denoted as baseline (BL). Increasing concentrations of heparin caused a rightward shift in the protamine concentration-response curves, both in peak calcium mobilization (A; quantified using the fluorescence dye, FURA-2) and degranulation (B; quantified by the release of *β*-hexosaminidase and calculated as a percentage of total cellular *β*-hexosaminidase). Highlighted in red are the data points that correspond to a protamine-heparin ratio of 1:1. N ≥ 5; mean ± SEM.

We next examined the actions of heparin on protamine-induced mast cell activation. In both Ca^2+^ mobilization and degranulation studies in LAD2 cells. As expected, heparin produced a concentration-dependent inhibition of protamine’s effects visualized by the rightward shift of the protamine concentration-response curve (Fig. 2). Interestingly, at the recommended clinical 1:1 ratio of protamine:heparin, equivalent to 10 *μ*g:1 USP unit (shown by symbols colored in red), there remained residual mast cell activation (Fig. 2, A and B).

These results are consistent with the visual observation of RPMC degranulation captured in real-time where heparin again inhibits protamine-induced degranulation (Supplemental Video 1B). Similar to the results using LAD2 WT cells where degranulation was measured through *β*-hexosaminidase release, there was no observable degranulation following protamine addition in the presence of an excess of heparin (Supplemental Video 1C).

### 3.3 Effect of heparin on other exogenous and endogenous MRGPRX2 agonists

**Fig. 3:**
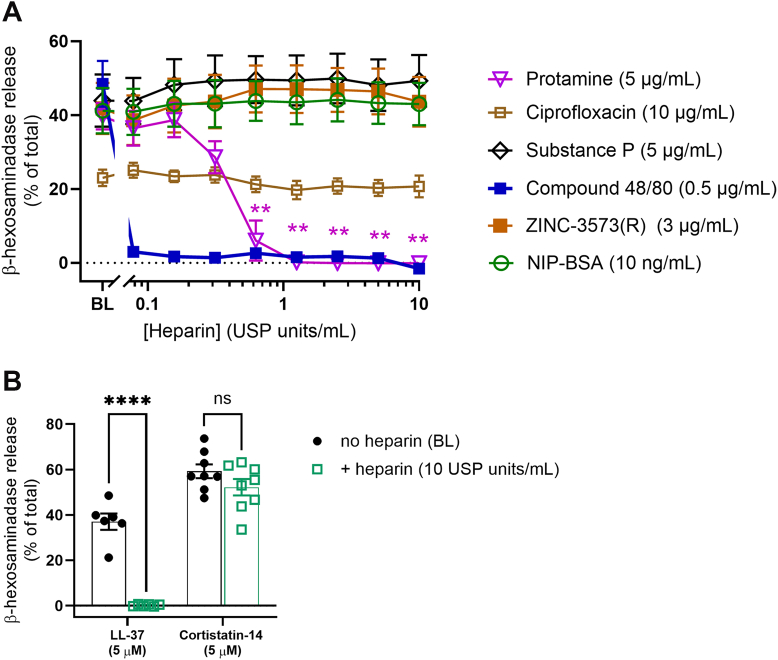
Heparin selectively inhibits MRGPRX2 agonist-induced degranulation. LAD2 cells were stimulated with a submaximal concentration of known mast cell and MRGPRX2 activators either independently or in complex with heparin (0.070–10 USP units/mL). Responses in the absence of heparin are denoted as baseline (BL). Degranulation was measured 45 minutes post stimulation by quantification of *β*-hexosaminidase release. (A) Increasing concentrations of heparin inhibited protamine (5 *μ*g/mL) and compound 48/80 (0.5 *μ*g/mL)-mediated LAD2 degranulation but had no effect on ZINC-3573(R) (3 μg/mL), substance P (5 *μ*g/mL), ciprofloxacin (200 *μ*g/mL) and NIP-BSA (10 ng/mL)-induced degranulation. N ≥ 5; mean ± SEM. One-way ANOVA with Dunnett’s multiple comparison post hoc test was conducted. ∗∗*P* < .01. (B) Heparin significantly inhibited LAD2 degranulation by LL-37 (5 *μ*M) but had no effect on the cortistatin-14 (5 *μ*M) response. N ≥ 6, mean ± SEM. For statistical analysis, a two-way ANOVA was conducted with Bonferroni multiple comparison post hoc test. ∗∗∗∗*P* < .0001.

Heparin acts by binding to polybasic regions in protamine. Therefore, we sought to determine if it could also antagonize other polybasic MRGPRX2 agonists. In these studies, a submaximal concentration of each stimulus was premixed with a range of heparin concentrations prior to LAD2 cell exposure. As expected, the protamine-induced LAD2 degranulation was significantly, and concentration-dependently, impaired when coincubated with heparin (Fig. 3A). Furthermore, degranulation induced by compound 48/80 was also effectively inhibited when coincubated with heparin (Fig. 3A), whereas LAD2 cell degranulation stimulated by the exogenous small molecule MRGPRX2 agonists, ZINC-3573(R), and ciprofloxacin (and the Fc*ε*RI /IgE pathway agonist NIP-BSA), were not affected by heparin (Fig. 3A). Heparin did attenuate MRGPRX2-dependent LAD2 cell degranulation triggered by the endogenous host defense peptide, LL-37, but not by the neuropeptide agonists cortistatin-14 and substance P (Fig. 3, A and B).

### 3.4 Characterization of MRGPRX2 agonist biding to heparin using

**Fig. 4:**
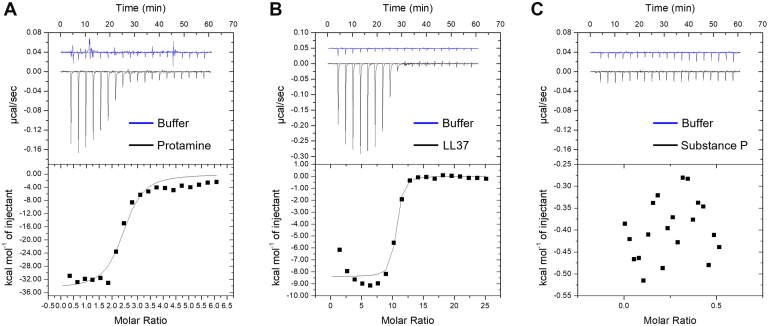
Heparin binds protamine and LL-37 but not substance P. ITC measurement of (A) protamine (200 *μ*g/mL), (B) LL37 (200 *μ*M), and (C) substance P (80 *μ*M) titrated against a fixed optimized concentrations of heparin (2, 4, 16 USP units/mL) (left to right) (shown in black), and buffer titrant control (shown in blue). Interaction analyses were fitted using a 1-site binding model and are shown as raw thermograms (top panel) and binding isotherms (bottom panel). No binding was seen between heparin and substance P. Representative data are shown from N = 3.

To help identify the mechanism behind the selectivity of MRGPRX2 ligand neutralization by heparin we conducted isothermal titration calorimetry (ITC) studies. These experiments revealed that protamine and LL37 bind strongly to heparin with high affinity, as indicated by estimated low nanomolar dissociation constants (44.8 ± 2.9 nM and 37.3 ± 3.5 nM). However, substance P showed no detectable binding to heparin or significant change in enthalpy, indicating minimal interaction under the experimental conditions tested (Fig. 4; Supplemental Table 2).

## 4 Discussion

We have demonstrated that human mast cell degranulation by the polybasic drug protamine can occur through direct activation of the MRGPRX2 receptor. We show that relative to the known full agonist of MRGPRX2, compound 48/80, protamine demonstrated proportionate activation across all measured pathways, but with lowered maximal efficacy, and is therefore characterized as a nonbiased, partial agonist at the receptor. The partial agonism of protamine sheds new light on the pharmacology of MRGPRX2, particularly in the context of diverse ligand interactions.

In keeping with stoichiometric chemical antagonism between the positively charged protamine and negatively charged heparin, agonism at MRGPRX2 was attenuated in a concentration-dependent manner by heparin. Although in vivo experiments have not been conducted to support these findings, our in vitro data indicate that in the clinical reversal of heparin, if protamine is in excess, there could be an increase in the likelihood of MRGPRX2 activation, and in susceptible individuals, potentially IgE-independent anaphylaxis. In a clinical setting, determining the dose of protamine to effectively and safely reverse the anticoagulant activity of heparin is inconsistent and continues to be debated.^26^ Although guidelines recommend administration of 1–1.5 mg protamine per 100 USP units of heparin,^1^^,^^27^, ^28^, ^29^ in practice, sequential measurements of activated clotting time further inform protamine dosing.^26^^,^^30^ In addition, there has been recent interest in managing cases of protamine anaphylaxis requiring rescue cardiopulmonary bypass,^2^ where the safety of protamine re-exposure following patient stabilization remains a concern.^31^

However, in vivo, given the large, polybasic nature of protamine and its intravenous route of administration, the relative proportion (if any) of protamine that could perfuse into extravascular tissues to trigger anaphylaxis by activating extravascular mast cells remain unclear. Nevertheless, mast cells are commonly located close to blood vessels and have been demonstrated to be activated through the IgE pathway by binding IgE intravascularly through the direct sampling of the intravascular lumen.^32^ In addition, blood antigens are known to be shuttled to mast cells via microvesicles released by perivascular dendritic cells.^33^ Thus, it is conceivable that such mechanisms might lead to mast cell activation by protamine. Upon this initial mast cell activation, the release of histamine and other mast cell mediators would increase local vascular permeability by disrupting endothelial barriers,^34^ thereby further increasing the likelihood of the originally vascular protamine encountering mast cells. In addition, protamine is also known to increase vascular permeability,^35^^,^^36^ thereby potentially also increasing extravascular mast cell exposure to the drug.

Given that the incidence of protamine anaphylaxis is relatively rare (0.6%–1% in a review study)^4^ and that we currently do not know the factors, pharmacogenomic or other, that render certain individuals more susceptible to drug-induced MRGPRX2-dependent mast cell activation, the importance of this mechanism to protamine-mediated clinically-manifested anaphylaxis is unclear. Nevertheless, although extrapolation of these in vitro results to the clinic should be done carefully, our data support the likely benefit of the careful use of protamine in neutralizing heparin’s activity to reduce the risk of IgE-independent anaphylaxis, as suggested by other studies.^26^^,^^28^^,^^37^ This extends not just to dose but also to the rate of protamine administration, which has additionally been associated with some cases of anaphylaxis.^7^

A large number of MRGPRX2 agonists have now been identified which span endogenous peptides like substance P and LL-37, therapeutic drugs such as ciprofloxacin and rocuronium, and laboratory tools such as compound 48/80 and ZINC-3573(R).^8^^,^^11^^,^^38^^,^^39^ Most MRGPRX2 agonists, like protamine, share a common property of being polybasic. Given our observation of heparin-mediated antagonism of protamine-induced MRGPRX2 activation, we extended our research to investigate the effect of heparin on other MRGPRX2 agonists. Heparin, as mentioned earlier, is a negatively charged glycosaminoglycan, and an abundant component of the mast cell granular matrix released upon degranulation.^40^ Heparin is known to have anti-inflammatory properties,^41^ and it is thus plausible that a component of this effect might relate to neutralization of endogenous MRGPRX2 agonists. Our data show that heparin inhibits MRGPRX2 activation by certain polybasic compounds, like compound 48/80 and the endogenous host defense peptide, LL-37, but had no effect on other known agonists including ZINC-3573(R), ciprofloxacin, and the endogenous neuropeptides substance P and cortistatin-14.

The charge/charge interaction between protamine and heparin has been well characterized.^1^^,^^29^ However, our results show this is not necessarily a universally simple interaction between heparin and other MRGPRX2 agonists but rather displays selectivity. Although complex formation with heparin may successfully mask the essential cationic binding sites of protamine and compound 48/80, preventing them from interacting with the specific MRGPRX2 amino acids necessary for receptor activation, the interaction of other basic stimuli with heparin might lack affinity, avidity, or may not fully mask the MRGPRX2-interacting sites of the agonist. Results from our ITC studies demonstrate that, at least for LL37 and substance P, binding to heparin is the key discriminator, with the latter MRGRX2 agonist exhibiting no measurable interaction with heparin. This suggests that selective binding and neutralization of endogenous polybasic MRGPRX2 agonists may underpin at least part of the observed anti-inflammatory actions of heparin.

In conclusion, this study has shown that protamine can directly activate human mast cells through MRGPRX2. Heparin was found to attenuate this response, highlighting the importance of the protamine/heparin ratio as a potential factor in the initiation of anaphylaxis in relevant clinical settings. Furthermore, we show that heparin can inhibit the action of some MRGPRX2 agonists but not others. Further studies are needed to determine if exogenous, or indeed endogenous, heparin might exert some of its actions (eg*,* anti-inflammatory) via neutralization of polybasic activators of MRGPRX2.

## Conflict of interest

The authors declare no conflicts of interest.

## Supplemental material

Supplementary Tables 1-2 and Supplementary Figure 1

Supplementary Video Caption

Supplementary Video 1A

Supplementary Video 1B

Supplementary Video 1C
