# Supplementary material for: Direct protamine activation of human mast cells is MRGPRX2-dependent and is modulated by heparin
Source: J Pharmacol Exp Ther. 2026 Mar 4;393(4):104313. doi: 10.1016/j.jpet.2026.104313 (PMC13197942; doi:10.1016/j.jpet.2026.104313)

**The Journal of Pharmacology and Experimental Therapeutics**

**Title**: Direct protamine activation of human mast cells is MRGPRX2-dependent and is modulated by heparin.

**Authors:** Nithya A. Fernandopulle, Jie Ding, Gavan Francis, Mark D. Hulett, Paul F. Soeding, Lauren T. May, Graham A. Mackay

**Supplemental Material (Tables and Figures for publication)**

**Supplemental Table 1:** Pharmacological evaluation of MRGPRX2 agonists in LAD2 cells. Mast cell activation was quantified for calcium mobilisation, degranulation and cytokine CCL2 release. Each response was normalised to that of the full agonist, compound 48/80. A four-parameter logistic curve was used to quantify pEC50 and Emax. The Black-Leff operational model of partial agonism was used to quantify pKA and Log(τ). A reparameterization of the Black-Leff operational model was used to quantify Log(τ/KA). Quantification of biased agonism involved normalisation Log(τ/KA) to the reference agonist, compound 48/80, to derive ΔLog(τ/KA) and then normalisation Log(τ/KA) to the reference pathway, calcium mobilisation, to derive Log(Bias).

|  | pEC50 | Emax | pKA | Log(τ) | Log(τ/KA) | ΔLog(τ/KA) | Log(Bias) |
| --- | --- | --- | --- | --- | --- | --- | --- |
| Calcium mobilization | | | | | | | |
| Compound 48/80 | 7.17 ± 0.06 | 105 ± 5 | N.D. | N.D. | 7.19 ± 0.06 | 0 |  |
| Protamine | 7.65 ± 0.07 | 84 ± 4 | 7.49 ± 0.22 | 0.33 ± 0.11 | 7.82 ± 0.13 | 0.63 ± 0.14 | 0 |
| Degranulation | | | | | | | |
| Compound 48/80 | 6.99 ± 0.04 | 99 ± 3 | N.D. | N.D. | 6.99 ± 0.04 | 0 |  |
| Protamine | 7.68 ± 0.07 | 54 ± 2 | 7.76 ± 0.11 | 0.08 ± 0.02 | 7.83 ± 0.10 | 0.84 ± 0.10 | 0.21 ± 0.17 |
| Cytokine release | | | | | | | |
| Compound 48/80 | 6.58 ± 0.06 | 102 ± 6 | N.D. | N.D. | 6.58 ± 0.07 | 0 |  |
| Protamine | 7.46 ± 0.27 | 15 ± 3 | 7.61 ± 0.35 | -0.51 ± 0.02 | 7.12 ± 0.36 | 0.54 ± 0 | -0.09 ± 0.39 |

**Supplemental Table 2:** Estimated thermodynamic parameters of the interaction between heparin and MRGPRX2 agonists obtained using ITC. Estimated Kd values, binding sites (N) and enthalpy (∆H) and entropy (∆S) calculations are shown as mean values ±SD from 3 independent experiments.

| Ligand | K_d_ (nM) | N (mol/mol) | ∆H kcal/mol | ∆S cal/mol/deg |
| --- | --- | --- | --- | --- |
| Protamine | 44.8 ± 2.9 | 2.16 ± 0.1 | -35.06 ± 2.73 | -90.50 ± 9.2 |
| LL37 | 37.3 ± 3.5 | 10.47 ± 0.33 | -8.33 ± 0.16 | 6.08 ± 0.58 |

**Supplemental Figure 1:** Protamine activation of MRGPRX2 leads to Gq α/βγ dissociation.

A BRET-based G protein activation assay was used to quantify activation of MRGPRX2 by protamine and compound 48/80 in transiently transfected HEK-293 cells. A) Transfection with Gq only. B) Transfection with Gq and MRGPRX2. Representative standard error bars are shown at a time point of 20 seconds. C) Area under the curve for the above BRET assays were calculated and graphed to assess statistical significance. N=4, mean ±SEM. For statistical analysis, a two-way ANOVA was conducted with Bonferroni multiple comparison post hoc test. ***p < 0.001, ****p <0.0001.


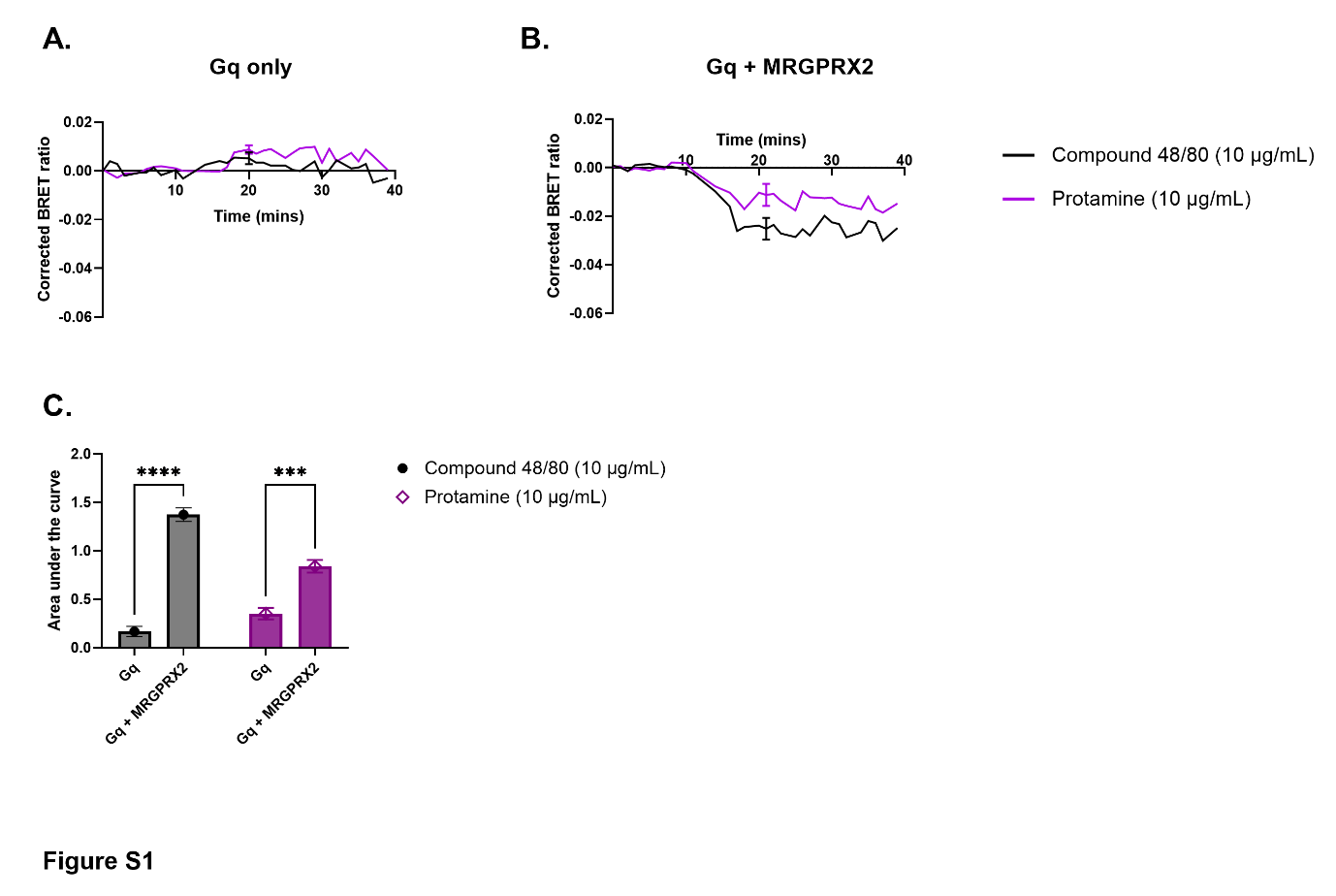

Supplement: Supplementary Tables 1-2 and Supplementary Figure 1 [file mmc1.docx]
