# Supplementary material for: Direct protamine activation of human mast cells is MRGPRX2-dependent and is modulated by heparin
Source: J Pharmacol Exp Ther. 2026 Mar 4;393(4):104313. doi: 10.1016/j.jpet.2026.104313 (PMC13197942; doi:10.1016/j.jpet.2026.104313)
Supplement: Supplementary Video Caption [file mmc2.docx]

**The Journal of Pharmacology and Experimental Therapeutics**

**Title**: Direct protamine activation of human mast cells is MRGPRX2-dependent and is modulated by heparin.

**Authors:** Nithya A. Fernandopulle, Jie Ding, Gavan Francis, Mark D. Hulett, Paul F. Soeding, Lauren T. May, Graham A. Mackay

**Supplemental Video 1 captions**

**Supplemental Video 1**: Real-time imaging of protamine-induced degranulation in rat peritoneal mast cells (RPMCs).

Live cells were imaged every 2 seconds for up to 60 seconds using a Leica SP5 confocal microscope and compiled into a short video using Fiji (version 1.42). Protamine (15 µg/mL)-induced RPMC degranulation (**Supplemental Video 1A**) was reduced in the presence of heparin at a ratio of 1:1 (15 µg/mL protamine: 1.5 USP units/mL heparin; **Supplemental Video 1B**). There was, however, no observed degranulation when the cells were exposed to a protamine:heparin ratio of 1:2 (15 µg/mL protamine: 3 USP units/mL heparin; **Supplemental Video 1C**).
